# Synergetic Association between Anemia and Hyperuricemia on New-Onset Chronic Kidney Disease in a Large Taiwanese Population Follow-Up Study

**DOI:** 10.3390/ijerph20021421

**Published:** 2023-01-12

**Authors:** You-Chi Chen, Yi-Hsueh Liu, Pei-Yu Wu, Jiun-Chi Huang, Ho-Ming Su, Szu-Chia Chen, Jer-Ming Chang

**Affiliations:** 1 Department of General Medicine, Kaohsiung Medical University Hospital, Kaohsiung Medical University, Kaohsiung 807, Taiwan; 2 Department of Internal Medicine, Kaohsiung Municipal Siaogang Hospital, Kaohsiung Medical University, Kaohsiung 812, Taiwan; 3 Division of Cardiology, Department of Internal Medicine, Kaohsiung Medical University Hospital, Kaohsiung 807, Taiwan; 4 Division of Nephrology, Department of Internal Medicine, Kaohsiung Medical University Hospital, Kaohsiung Medical University, Kaohsiung 807, Taiwan; 5 Faculty of Medicine, College of Medicine, Kaohsiung Medical University, Kaohsiung 807, Taiwan; 6 Research Center for Precision Environmental Medicine, Kaohsiung Medical University, Kaohsiung 807, Taiwan

**Keywords:** anemia, hyperuricemia, new-onset chronic kidney disease, Taiwan Biobank, follow-up, synergetic effect

## Abstract

The incidence of chronic kidney disease (CKD) is increasing worldwide; however, the association between CKD and anemia and hyperuricemia has yet to be clarified. In addition, whether anemia and hyperuricemia only influence renal damage in combination with other comorbidities or whether they are direct causative factors is also controversial. Therefore, the aim of this longitudinal study was to investigate these issues in a large Taiwanese cohort. We enrolled 26,631 participants from the Taiwan Biobank (TWB) after excluding those with CKD at the baseline, all of whom had follow-up data for a median of 4 years. In this study, CKD was defined as an estimated glomerular filtration rate < 60 mL/min/1.73 m^2^, incident new-onset CKD was defined as the development of CKD during follow-up, anemia was defined as a hemoglobin level <13 mg/dL in males and <12 mg/dL in females, and hyperuricemia was defined as a serum uric acid (UA) level >7 mg/dL in males and >6 mg/dL in females. The participants were divided into four groups according to whether or not they had anemia and hyperuricemia. Multivariable analysis showed that low hemoglobin (per 1 g/dL; odds ratio [OR], 0.760; *p* < 0.001) and high serum UA (per 1 mg/dL; OR, 1.444; *p* < 0.001) in model 1 and anemia (OR, 2.367; *p* < 0.001) and hyperuricemia (OR, 2.516; *p* < 0.001) in model 2 were significantly associated with new-onset CKD. Furthermore, compared to the group without anemia or hyperuricemia, the groups with anemia without hyperuricemia (OR, 2.502; *p* < 0.001), without anemia with hyperuricemia (OR, 2.559; *p* < 0.001), and with anemia and hyperuricemia (OR, 5.505; *p* < 0.001) were significantly associated with new-onset CKD. There was a significant interaction between hemoglobin and serum UA and new-onset CKD (*p* < 0.001). In conclusion, we found that anemia and hyperuricemia were associated with new-onset CKD, respectively, and also had a synergetic effect on new-onset CKD.

## 1. Introduction

The incidence and prevalence of chronic kidney disease (CKD) and end-stage renal disease (ESRD) are increasing worldwide, of which the prevalence of ESRD is highest in Taiwan [1,2]. The Taiwan Society of Nephrology reported that the incidence and prevalence of dialysis increased from 529/million in 2000 to 3679/million population in 2019, and that this was associated with high medical costs (8–9%) [3]. ESRD has been shown to increase the risk of cardiovascular events and death [4], and a fast decline in renal function has been reported to increase the risk of complications [5]. Consequently, the early detection of factors associated with CKD is crucial.

Anemia is a blood disorder in which the blood has a reduced ability to carry oxygen due to a lower number of red blood cells (RBCs) or a reduction in the amount of hemoglobin [6]. Anemia is common in patients with CKD, and it can cause symptoms including dyspnea, dizziness, weakness, and even cardiovascular events or conscious disturbance [7]. The pathogenesis of CKD-related anemia includes iron deficiency, insufficient erythropoietin production, erythropoietin hypo-responsiveness, decreased RBC half-life, and chronic inflammation [8,9]. Hyperuricemia is another comorbidity of CKD. Uric acid (UA) is the end product of purine catabolism in humans, and around two thirds of UA is excreted in urine [10,11]. In CKD patients, the lower glomerular filtration rate (GFR) results in decreased excretion of UA [10]. In addition, hyperuricemia associated with CKD and hypertension may be related to activation of the renin–angiotensin–aldosterone system, and the deposition of urate crystals can also cause renal tubular damage [10].

Although anemia and hyperuricemia are common comorbidities of CKD, whether they influence renal damage in combination with other comorbidities or whether they are direct causative factors is controversial. Anemia and hyperuricemia share several comorbidities; however, the association between the two conditions remains unclear. Eun et al. [12] investigated the association between anemia and hyperuricemia in 10,794 subjects from the Korean National Health and Nutrition Examination Survey conducted in 2016–2017 and found that an association between anemia and hyperuricemia was not evident in subjects without CKD, but that it was evident in those with CKD. In the patients with CKD, anemia increased the risk of hyperuricemia by 2-fold. Few studies have investigated the association between a combination of anemia and hyperuricemia and new-onset CKD. Therefore, the aim of this study was to investigate the associations among anemia, hyperuricemia, and their combination with new-onset CKD in a large cohort of Taiwanese adults.

## 2. Materials and Methods

### 2.1. Taiwan Biobank (TWB)

The TWB was launched by the Taiwan government in 2012 as an ongoing prospective study of men and women aged 30–70 years recruited from approximately 30 centers around the country to promote epidemiological and biomedical research. For each enrollee in the TWB, comprehensive genomic and phenotypic data are collected and recorded at enrollment and during follow-up through urine and blood tests, physical examination, and structured questionnaires. All participants in the TWB provide written informed consent before enrollment [13,14].

### 2.2. Laboratory, Demographic, and Medical Variables

The following demographic and medical data were collected: body mass index (BMI), age, sex, smoking and alcohol history, and medical history (hypertension and diabetes mellitus [DM]). The following laboratory data were also collected after an overnight fast: glucose, triglycerides, hemoglobin, UA, cholesterol (total, high-, and low-density lipoprotein [HDL/LDL]), and estimated GFR [eGFR] (calculated using the 4-variable Modification of Diet in Renal Disease study equation [15]).

Trained personnel performed blood pressure (BP) measurements (systolic and diastolic) digitally with the participants abstaining from exercise, caffeine, and smoking for at least 30 min before the measurements. Each measurement was made three times with a 1–2 min break between measurements; the average values were used for analysis.

### 2.3. Study Participants

**Figure 1:**
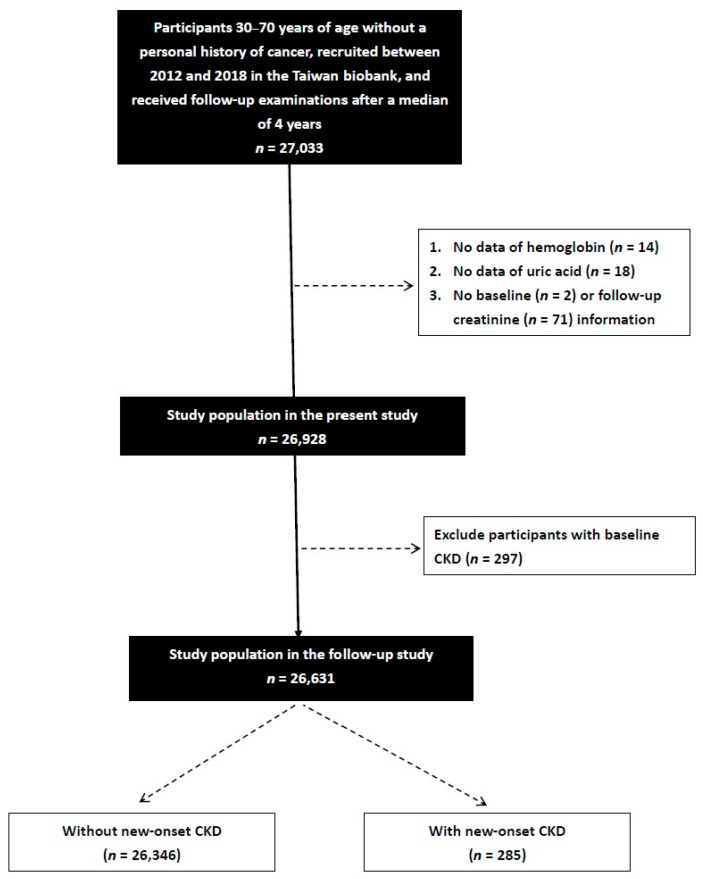
Flowchart of study population.

Between 2012 and 2018, a total of 27,033 TWB enrollees received follow-up examinations after 2–4 years. Information, including a questionnaire, physical examination, and blood examination, is collected upon first enrollment and second follow-up. The participants who did not have data on hemoglobin (*n* = 14), serum UA (*n* = 18), and creatinine (*n* = 73) were excluded. We further excluded those with CKD (eGFR < 60 mL/min/1.73 m^2^) at the baseline (*n* = 297), and the remaining 26,631 participants were enrolled (Figure 1).

### 2.4. Definition of New-Onset CKD

CKD was defined as an eGFR < 60 mL/min/1.73 m^2^. The participants with an eGFR greater than this value were deemed to not have CKD. Those who then developed CKD during follow-up were classified as the new-onset CKD group.

### 2.5. Definitions of Anemia and Hyperuricemia

Anemia was defined as a hemoglobin level <13 mg/dL in males and <12 mg/dL in females. Hyperuricemia was defined as a serum UA level >7 mg/dL in males and >6.0 mg/dL in females [16].

### 2.6. Ethics Statement

The Institutional Review Board (IRB) of Kaohsiung Medical University Hospital approved this study (KMUHIRB-E(I)-20210058). Ethical approval for the TWB was granted by the IRB on Biomedical Science Research, Academia Sinica, Taiwan, and the Ethics and Governance Council of the TWB. In addition, the study was conducted according to the Declaration of Helsinki.

### 2.7. Statistical Analysis

SPSS was used for statistical analysis (version 19 for Windows^®^, IBM Inc., Armonk, NY, USA). Data are shown as percentages or means ± SD as appropriate. The study participants were classified into four groups according to whether or not they had anemia and/or hyperuricemia. Among-group comparisons were performed using one-way analysis of variance followed by a Bonferroni-adjusted post-hoc test. Differences between categorical variables were analyzed using the chi-square test. Binary logistic regression analysis was used to identify factors associated with new-onset CKD, with significant variables entered into the multivariable analysis. The group without anemia or hyperuricemia was taken as the reference due to it having the smallest incidence rate. In the logistic analysis, an interaction *p* was defined as: model disease (y) = x1 + x2 + x1 * x2 + covariates, where x1 * x2 is the interaction term, y is the new-onset CKD, x1 is the hemoglobin, and x2 is the serum UA. The covariates were significant variables in the univariable analysis. A *p* value < 0.05 was considered significant.

## 3. Results

Among the 26,631 enrolled participants (9355 males, 17,276 females), the mean age was 51.1 ± 10.4 years. The four groups were those with both anemia and hyperuricemia, those with anemia without hyperuricemia, those with hyperuricemia without anemia, and those without either anemia or hyperuricemia.

### 3.1. Comparisons of Clinical Characteristics among the Four Study Groups

Compared to the group without anemia or hyperuricemia, the group with anemia and hyperuricemia had a higher rate of hypertension, higher systolic BP, higher BMI, lower hemoglobin, higher serum UA, higher triglycerides, lower total cholesterol, lower HDL-cholesterol, lower LDL-cholesterol, and lower eGFR (Table 1). With regards to the outcomes, the group with anemia and hyperuricemia had the highest prevalence of new-onset CKD among the four study groups (*p* ≤ 0.004).

### 3.2. Factors Associated with New-Onset CKD

The factors associated with new-onset CKD in the univariable logistic regression analysis were older age, male sex, smoking and alcohol history, DM, hypertension, high systolic and diastolic BPs, high BMI, high fasting glucose, high triglycerides, and low HDL-cholesterol (Table 2). However, total cholesterol and LDL-cholesterol were not associated with new-onset CKD.

### 3.3. Associations of Hemoglobin, UA, Anemia, Hyperuricemia, and Study Groups with New-Onset CKD

Table 3 shows the associations of hemoglobin, UA, anemia, hyperuricemia, and study groups with new-onset CKD using multivariable logistic regression analysis. After adjusting for hemoglobin, UA, and the significant variables (age, sex, smoking and alcohol history, DM, hypertension, systolic and diastolic BPs, BMI, fasting glucose, triglycerides, and HDL-cholesterol) in Table 1 (model 1), low hemoglobin (per 1 g/dL; odds ratio [OR], 0.760; 95% confidence interval [CI], 0.689–0.837; *p* < 0.001), high serum UA (per 1 mg/dL; OR, 1.444; 95% CI, 1.323–1.576; *p* < 0.001), old age, male sex, alcohol history, DM, hypertension, and high systolic BP, BMI, fasting glucose, and triglycerides were significantly associated with new-onset CKD. Moreover, a significant interaction between hemoglobin and serum UA and new-onset CKD was found in the interaction analysis (*p* < 0.001).

In addition, after adjusting for anemia, hyperuricemia, and the significant variables in Table 1 (model 2), anemia (OR, 2.367; 95% CI, 1.625–6.448; *p* < 0.001), hyperuricemia (OR, 2.516; 95% CI, 1.952–3.244; *p* < 0.001), older age, male sex, alcohol history, DM, hypertension, and high systolic BP, BMI, fasting glucose, and triglycerides were significantly associated with new-onset CKD (Table 3).

Finally, after adjusting for the four study groups and the significant variables in Table 1 (model 3), the group with anemia without hyperuricemia (vs. without anemia or hyperuricemia; OR, 2.502; 95% CI, 1.571–3.987; *p* < 0.001), the group without anemia with hyperuricemia (vs. without anemia or hyperuricemia; OR, 2.559; 95% CI, 1.957–3.345; *p* < 0.001), the group with anemia and hyperuricemia (vs. without anemia or hyperuricemia; OR, 5.505; 95% CI, 2.955–10.254; *p* < 0.001), older age, male sex, alcohol history, DM, hypertension, and high systolic BP, BMI, fasting glucose, and triglycerides were significantly associated with new-onset CKD (Table 3).

## 4. Discussion

In this study, we investigated the associations between a combination of anemia and hyperuricemia and new-onset CKD after a median 4-year follow-up period. We found that anemia (defined as a hemoglobin level <13 mg/dL in males and <12 mg/dL in females) and hyperuricemia (defined as a serum UA level >7 mg/dL in males and >6.0 mg/dL in females) were associated with new-onset CKD, respectively. There was also a significant interaction between hemoglobin and serum UA and new-onset CKD, and a synergetic effect of anemia and hyperuricemia on new-onset CKD was also noted.

The first important result is the association between low hemoglobin and anemia and new-onset CKD. Anemia has been reported to be an independent risk factor for kidney disease progression to ESRD or mortality [17,18]. The mechanism potentially underlying anemia-induced accelerated renal function progression may be hypoxia. Hypoxia may reduce the oxygen supply to tubular cells, subsequently leading to nephron loss through chronic ischemia [19,20]. Siems et al. [21] showed that serum levels of aldehydic lipid peroxidation (LPO) products and protein carbonyls, which were analyzed as parameters of oxidative stress, were higher in chronic hemodialysis patients than in healthy people. Moreover, the stronger the degree of anemia, the higher the serum levels of aldehydic LPO products and protein carbonyls, resulting in endothelial dysfunction and glomerular basement membrane damage [21,22]. Another mechanism for the association between anemia and CKD may be related to the oxidative stress induced by anemia. Iron-deficiency anemia, a common type of anemia in patients with CKD, is caused by iron and erythropoietin deficiencies and a erythropoietin hypo-responsiveness, which reduces the lifespan of RBCs and enhances the risk of oxidative stress [22]. We suggest that anemia is not only a complication of CKD, but that it may also be a cause of CKD.

Another important finding is that high UA and hyperuricemia were associated with new-onset CKD. An elevated serum UA level has been reported to be an independent predictor of CKD development in several studies. Iseki et al. reported a relationship between new-onset CKD and elevated UA in 6403 patients [23]. In addition, they found that a UA level of 8.0 mg/dL was associated with a 2.9-fold increased risk of an elevated serum creatinine level in males and 10.4-fold increased risk in females after controlling for other risk factors [23]. In addition, Obermayr et al. reported that an association between a modest elevation in UA level (7.0–8.9 mg/dL) was associated with an new-onset CKD in 21,475 healthy volunteers followed prospectively for 7 years with an OR of 1.74 (95% CI 1.45 to 2.09), compared to 3.12 (95% CI 2.29 to 4.25) for a marked elevation in UA level (≥9.0 mg/dL) [24]. Moreover, in an analysis of pooled data from the Atherosclerosis Risk in Communities (ARIC) Study and Cardiovascular Health Study (CHS), Weiner et al. reported that a higher serum UA level was modestly independently associated with a decline in renal function (OR 1.07; 95% CI 1.01 to 1.14) [25]. A previous animal experiment also showed that elevated serum UA was associated with an increased serum creatinine level and the risk of glomerulosclerosis and interstitial fibrosis development [26]. Furthermore, another study reported that renal injury could be avoided with the use of xanthine oxidase inhibitors to prevent hyperuricemia [27]. The mechanism appears to be induction of arteriolopathy, which exacerbates glomerular hypoxia due to the accumulation of reactive oxygen species [28,29]. Another study reported that increased serum UA was significantly and inversely associated with ultrasonographic flow-mediated dilatation, indicating that endothelial dysfunction was correlated with high UA levels [30]. Moreover, another study [24] demonstrated a higher risk of incident kidney disease in patients with a serum UA level above 6 to 7 mg/dL in women and 7 to 8 mg/dL in men, which is compatible with the results of our study. Furthermore, the deposition of UA crystals in renal tubules can obstruct the tubules, and induce local inflammation and interstitial fibrosis [31]. A link between elevated UA and hypertension [32,33,34], diabetes mellitus [35,36], and atherosclerosis [37,38] has been reported in previous epidemiological studies, and these factors may contribute to CKD development.

We also found a significant interaction between hemoglobin and serum UA and new-onset CKD, and a synergetic effect of anemia and hyperuricemia on new-onset CKD. We classified the patients into four groups according to whether or not they had anemia and/or hyperuricemia, and found that the group with both anemia and hyperuricemia had a higher OR of 5.505. Several studies have investigated the association between UA or hemoglobin and renal function; however, we investigated the synergistic effect of both and found an association with CKD. Both anemia [39,40] and hyperuricemia [41] have been shown to induce oxidative stress and inflammation in the extracellular matrix and endothelial cells, resulting in glomerular hypertension and sclerosis. This could then impair blood flow to the kidneys, increase vascular resistance in the kidneys, and lead to a decline in eGFR. Moreover, a decline in renal function may progressively impair the metabolism and clearance of UA and anemia. Our findings imply that the synergistic effect of anemia and hyperuricemia may further impair renal function and eGFR.

A strength of this study is the large participant cohort and the high proportion of participants with complete follow-up data for the association of anemia and hyperuricemia with new-onset CKD. However, there were still a few limitations. First, data on anti-hypertensive, anti-diabetic, lipid-lowering, or urate-lowering drugs are not recorded in the TWB, and these agents could influence fasting glucose, serum hemoglobin, lipid profiles, BP, UA, and the occurrence of new-onset CKD, as well as the associations among anemia, hyperuricemia, and new-onset CKD. Second, we did not survey the association between different subtypes of anemia and new-onset CKD, or the potential risk factors causing a rapid worsening of renal function, such as proteinuria, which may have influenced the study results. Third, since our participants were all of Chinese ethnicity, the generalizability of our results to other groups needs to be clarified. Fourth, in the multivariate analysis, selecting the independent variable from the statistically significant factors in the univariate analysis may not be appropriate [42]. However, we tried to use multivariable forward analysis (included all variables of Table 2) of Table 3 and found similar results. Finally, the number of serum creatinine measurements was twice, and the interval was varied in each participant. Therefore, the exact time of new-onset CKD was unknown. Further longitudinal studies are needed to validate this issue.

## 5. Conclusions

In conclusion, we found that anemia and hyperuricemia were associated with new-onset CKD, respectively, and that anemia and hyperuricemia had a synergetic effect on new-onset CKD in this large Taiwanese adult follow-up study. Assessments of serum hemoglobin and UA level may help to identify those at high risk of CKD, and improved management of anemia and hyperuricemia may help to reduce the risk of developing CKD.

## Figures and Tables

**Table 1:** Comparison of clinical characteristics among four study groups according to anemia and hyperuricemia.

Characteristics	Without Anemia and Hyperuricemia (*n* = 18,712)	With Anemia, without Hyperuricemia (*n* = 2580)	Without Anemia, with Hyperuricemia (*n* = 5057)	With Anemia and Hyperuricemia (*n* = 282)	*p*
Age (year)	51.3 ± 10.3	47.6 ± 10.0 *	51.9 ± 10.5 *^#^	52.1 ± 10.4 ^#^	<0.001
Male (%)	33.4	10.8 *	54.2 *^#^	29.1 ^#†^	<0.001
Smoking history (%)	24.0	13.2 *	37.5 *^#^	21.6 ^#†^	<0.001
Alcohol history (%)	2.5	1.6	5.2 *^#^	1.8 ^†^	<0.001
DM (%)	5.0	3.6 *	6.0 *^#^	8.5 *^#^	<0.001
Hypertension (%)	11.2	6.4 *	20.9 *^#^	22.7 *^#^	<0.001
Systolic BP (mmHg)	116.7 ± 17.5	111.0 ± 16.5 *	123.1 ± 16.8 *^#^	121.6 ± 18.1 *^#^	<0.001
Diastolic BP (mmHg)	72.0 ± 10.6	67.4 ± 9.9 *	76.7 ± 10.5 *^#^	72.9 ± 11.4 ^#†^	<0.001
BMI (kg/m^2^)	23.6 ± 3.3	22.9 ± 3.2 *	26.2 ± 3.7 *^#^	25.9 ± 3.9 *^#^	<0.001
Laboratory parameters					
Hemoglobin (g/dL)	14.0 ± 1.2	11.0 ± 1.2 *	14.5 ± 1.2 *^#^	11.4 ± 1.0 *^#†^	<0.001
Uric acid (mg/dL)	5.0 ± 1.0	4.5 ± 0.9 *	7.5 ± 1.0 *^#^	7.2 ± 1.0 *^#†^	<0.001
Fasting glucose (mg/dL)	96.0 ± 20.8	91.8 ± 17.8 *	98.5 ± 17.3 *^#^	96.9 ± 17.6 ^#^	<0.001
Triglyceride (mg/dL)	106.6 ± 75.9	87.9 ± 60.3 *	151.8 ± 103.5 *^#^	133.8 ± 78.9 *^#†^	<0.001
Total cholesterol (mg/dL)	195.8 ± 34.7	181.2 ± 32.7 *	201.6 ± 36.7 *^#^	186.3 ± 35.4 *^†^	<0.001
HDL-cholesterol (mg/dL)	55.7 ± 13.3	56.2 ± 12.9	48.7 ± 11.3 *^#^	47.7 ± 10.5 *^#^	<0.001
LDL-cholesterol (mg/dL)	121.6 ± 31.0	109.5 ± 28.6 *	128.2 ± 33.1 *^#^	116.0 ± 31.3 *^#†^	<0.001
eGFR (mL/min/1.73 m^2^)	111.9 ± 24.1	119.3 ± 27.1 *	97.9 ± 20.3 *^#^	103.7 ± 28.4 *^#†^	<0.001
Outcome					
New-onset CKD (%)	0.66	0.89	2.47 *^#^	4.61 *^#†^	<0.001
Follow-up period (year)	3.8 ± 1.1	3.9 ± 1.2 *	3.8 ± 1.1 ^#^	3.8 ± 1.1	0.002

Abbreviations: DM, diabetes mellitus; BP, blood pressure; BMI, body mass index; HDL, high-density lipoprotein; LDL, low-density lipoprotein; eGFR, estimated glomerular filtration rate. Anemia is defined as participants with hemoglobin <13 mg/dL in males and <12 mg/dL in females. Hyperuricemia is defined as participants with serum uric acid >7 mg/dL in males and >6 mg/dL in females. * *p* < 0.05 compared with group without anemia and hyperuricemia; ^#^ *p* < 0.05 compared with group with anemia, without hyperuricemia; and ^†^ *p* < 0.05 compared with group without anemia, with hyperuricemia.

**Table 2:** Determinants for new-onset CKD using univariable logistic regression analysis.

Parameters	New-Onset CKD		
	Univariable		
	OR	95% CI	*p*
Age (per 1 year)	1.109	1.092–1.127	<0.001
Female (vs. male)	0.341	0.269–0.434	<0.001
Smoking history	2.118	1.671–2.684	<0.001
Alcohol history	3.160	2.067–4.830	<0.001
DM	5.878	4.438–7.784	<0.001
Hypertension	5.076	4.505–7.228	<0.001
Systolic BP (per 1 mmHg)	1.042	1.036–1.047	<0.001
Diastolic BP (per 1 mmHg)	1.043	1.033–1.054	<0.001
BMI (per 1 kg/m^2^)	1.138	1.109–1.168	<0.001
Laboratory parameters			
Fasting glucose (per 1 mg/dL)	1.015	1.012–1.017	<0.001
Triglyceride (per 1 mg/dL)	1.003	1.002–1.003	<0.001
Total cholesterol (per 1 mg/dL)	1.001	0.997–1.004	0.657
HDL-cholesterol (per 1 mg/dL)	0.961	0.951–0.971	<0.001
LDL-cholesterol (per 1 mg/dL)	0.998	0.994–1.002	0.351

Values expressed as odds ratio (OR) and 95% confidence interval (CI). Abbreviations are the same as in Table 1.

**Table 3:** Relation of hemoglobin, uric acid, anemia, hyperuricemia, and study groups and new-onset CKD using multivariable logistic regression analysis.

Parameters	Model 1		Model 2		Model 3	
	OR (95% CI)	*p*	OR (95% CI)	*p*	OR (95% CI)	*p*
Hemoglobin (per 1 g/dL)	0.760 (0.689–0.837)	<0.001	--	--	--	--
Uric acid (per 1 mg/dL)	1.444 (1.323–1.576)	<0.001	--	--	--	--
Anemia	--	--	2.367 (1.625–6.448)	<0.001	--	--
Hyperuricemia	--	--	2.516 (1.952–3.244)	<0.001	--	--
Study groups						
Without anemia and hyperuricemia	--	--	--	--	Reference	
With anemia, without hyperuricemia	--	--	--	--	2.502 (1.571–3.987)	<0.001
Without anemia, with hyperuricemia	--	--	--	--	2.559 (1.957–3.345)	<0.001
With anemia and hyperuricemia	--	--	--	--	5.505 (2.955–10.254)	<0.001
Age (per 1 year)	1.079 (1.060–1.098)	<0.001	1.080 (1.061–1.099)	<0.001	1.080 (1.061–1.099)	<0.001
Female (vs. male)	0.475 (0.331–0.681)	<0.001	0.491 (0.355–0.679)	<0.001	0.490 (0.354–0.677)	<0.001
Smoking history	1.083 (0.795–1.476)	0.611	1.094 (0.804–1.491)	0.567	1.092 (0.802–1.488)	0.575
Alcohol history	2.067 (1.298–3.290)	0.002	2.027 (1.274–3.225)	0.003	2.027 (1.274–3.224)	0.003
DM	1.733 (1.222–2.457)	0.002	1.776 (1.254–2.515)	0.001	1.778 (1.256–2.518)	0.001
Hypertension	1.696 (1.293–2.225)	<0.001	1.745 (1.331–2.287)	<0.001	1.745 (1.331–2.287)	<0.001
Systolic BP (per 1 mmHg)	1.021 (1.011–1.030)	<0.001	1.022 (1.012–1.031)	<0.001	1.022 (1.012–1.031)	<0.001
Diastolic BP (per 1 mmHg)	0.996 (0.980–1.012)	0.608	0.991 (0.976–1.007)	0.279	0.991 (0.976–1.007)	0.278
BMI (per 1 kg/m^2^)	1.056 (1.019–1.095)	0.003	1.061 (1.024–1.099)	0.001	1.061 (1.024–1.099)	<0.001
Fasting glucose (per 1 mg/dL)	1.010 (1.006–1.013)	<0.001	1.008 (1.004–1.012)	<0.001	1.008 (1.004–1.012)	<0.001
Triglyceride (per 1 mg/dL)	1.002 (1.001–1.003)	<0.001	1.002 (1.001–1.003)	<0.001	1.002 (1.001–1.003)	<0.001
HDL-cholesterol (per 1 mg/dL)	1.003 (0.992–1.015)	0.605	1.002 (0.990–1.013)	0.791	1.002 (0.990–1.013)	0.788

Values expressed as odds ratio (OR) and 95% confidence interval (CI). Abbreviations are the same as in Table 1. Anemia is defined as participants with hemoglobin < 13 mg/dL in males and <12 mg/dL in females. Hyperuricemia is defined as participants with serum uric acid > 7 mg/dL in males and >6 mg/dL in females. Multivariate model 1: adjusted for hemoglobin, uric acid, age, sex, smoking and alcohol history, DM, hypertension, systolic and diastolic BPs, BMI, fasting glucose, triglycerides, and HDL-cholesterol (significant variable in Table 1). Multivariate model 2: adjusted for anemia, hyperuricemia, age, sex, smoking and alcohol history, DM, hypertension, systolic and diastolic BPs, BMI, fasting glucose, triglycerides, and HDL-cholesterol (significant variable in Table 1). Multivariate model 3: adjusted for four study groups, age, sex, smoking and alcohol history, DM, hypertension, systolic and diastolic BPs, BMI, fasting glucose, triglycerides, and HDL-cholesterol (significant variable in Table 1).

## Data Availability

The data underlying this study are from the Taiwan Biobank. Due to restrictions placed on the data by the Personal Information Protection Act of Taiwan, the minimal data set cannot be made publicly available. Data may be available upon request to interested researchers. Please send data requests to: Szu-Chia Chen, PhD, MD. Division of Nephrology, Department of Internal Medicine, Kaohsiung Medical University Hospital, Kaohsiung Medical University.
